# Culture, church, and collective: a qualitative study about gambling harm prevention and reduction in Aotearoa/New Zealand—a Tongan male perspective

**DOI:** 10.1186/s12954-022-00717-2

**Published:** 2022-12-03

**Authors:** Edmond S. Fehoko, Maria E. Bellringer, Peggy Fairbairn-Dunlop

**Affiliations:** 1 Department of Human Nutrition, Division of Sciences, University of Otago, Dunedin, New Zealand; 2 Gambling and Addiction Research Centre, Auckland University of Technology, Auckland, New Zealand; 3 School of Social Sciences and Public Policy, Faculty of Culture and Society, Auckland University of Technology, Auckland, New Zealand

**Keywords:** Gambling, Talanoa, Cultural, Community, Church, Gambling harm providers

## Abstract

**Background:**

In New Zealand, Pacific people continue to be more at risk of gambling harm than the general population, despite increasing public health efforts and treatment service provisions introduced to address this social and health issue. In looking at why this is so, our first concern was to ask why the delivery of the prevailing gambling-focussed programmes was not influencing Pacific gambling behaviours. In seeking to answer this question, it was important to explore ethnic-Pacific-specific factors of gambling harm prevention and reduction.

**Methods:**

The research design was interpretivist/constructivist and phenomenological, applied through the lens of a Tongan worldview. Participants comprised Tongan male elders and youth. Recruitment of participants was through snowball sampling from churches and kava-drinking circles. A total of 28 elders and 18 youth participated through focus group talanoa and individual talanoa. This study employed descriptive thematic analysis.

**Results:**

Participants were not aware of any policy document or problem-gambling preventative programmes. Four key themes were raised, which include raising the awareness of existing gambling harm treatment providers, the church influence in addressing gambling harm, community-based strategies, and cultural-based approaches.

**Conclusions:**

This study proposes several recommendations such as more awareness of gambling harm providers in community, increasing cultural spaces and church engagements, and calls for further research in addressing the prevention and reduction of gambling harm amongst the Tongan community in New Zealand.

## Introduction

Most people in New Zealand have engaged in some form of gambling or know of somebody who has [1]. Gambling harm remains a significant public health and social issue for individuals, families, and communities. However, that harm is not evenly spread across New Zealand individuals, families, and communities [1, 2]. In fact, for the past 30 years, Pacific people have continued to have a high prevalence of gambling and gambling harm in New Zealand [3, 4]. This is despite the steady increase in gambling prevention and reduction information and providers available for the Pacific community [5, 6].

Pacific people were 2.56 times more likely to be moderate-risk or problem gamblers than non-Māori (New Zealand’s indigenous population) and non-Pacific people. An estimated 3.0 per cent of Pacific adults were moderate-risk or problem gamblers, and 4.4 per cent were low-risk gamblers. (Ministry of Health, 2022). Furthermore, Pacific people are more likely to be at risk of future harm than other ethnic groups (Problem Foundation of New Zealand, 2020). Bellringer and colleagues (2019) found that one in five Pacific youth were worried about the level of gambling at home, and one in nine experienced at least one household problem from that gambling. It is important to note that there is a dearth of quantitative and qualitative research on gambling and gambling harm relating to Pacific people in New Zealand, and there is limited ethnic-Pacific-specific gambling and gambling harm research.

In New Zealand, legislation was passed and policy was established to reduce the development of gambling harm. The Gambling Act 2003 defines harm as “any kind of harm or distress arising from, or caused or exacerbated by, a person’s gambling” [7]. This definition includes the psychological or emotional impacts of gambling, as well as the presumably more concrete forms of harm such as financial loss. This is emphasised in the second part of the definition, which explicitly refers to personal, social, or economic harms. The New Zealand definition also emphasises the multiple social scales at which harm can take place, which is more consistent with a social model of health, enumerating four levels at which harm may occur: the individual person; spouse, family, whānau (extended family), or wider community; in the workplace; or in society at large. A total of $24.84 million has been budgeted for public health services focussing on the reduction and prevention of gambling problems in the three-year period from 2022/23 to 2024/25 [1]. This three-year service plan by the Ministry of Health [1] proposed to:

*Continue providing dedicated services for Māori, and for Pacific and Asian people, where appropriate, including services both for gamblers and for their families; continue to ensure that the provision of all services to prevent and minimise gambling harm is culturally appropriate; and ensure that all services are health literate, high quality and effective. (p. 20)*

Studies have found that unfamiliarity can act as a barrier preventing Pacific people accessing some health services [8, 9]. Research by Ludeke and colleagues [10] argued that the presence of Pacific health professionals was a positive influence for members of the Pacific community to seek primary care. Other research, however, illustrated that some Pacific people have concerns regarding accessing care or support from a Pacific worker [11]. This is largely due to the small size of the Pacific community and the risk of jeopardised confidentiality [12]. Given that the diversity of New Zealand’s Pacific population and disproportionate levels of gambling harm Pacific people were facing, it is important to understand why Pacific people continue to be disproportionately affected by gambling harm. To address this topic, we used an ethnic-specific approach to conduct an in-depth qualitative study. Our focus was Tongan males, given their central place in Tonga’s hierarchical and monarchical systems and as head of the family, and their role in holding and passing on family knowledge. The first author [EF] is of Tongan descent, is fluent in the Tongan language, and is well respected in the Tongan community across Aotearoa/New Zealand. To that point, talanoa was employed for this study as the most appropriate research tool, particularly on sensitive issues such as gambling. As part of the first author’s doctoral study, this paper captures the perceptions and views of Tongan males which addressed his third research question:

1. What strategies can minimise or prevent the development of problem-gambling behaviours in New Zealand’s Tongan community?

## Methods

This study employed an interpretivist/constructivist approach as this offered participants the opportunity to connect and engage in multiple ways of knowing and knowledge building on the topic of gambling. Furthermore, the process of bringing together their views enabled a co-construction in the creation of knowledge, also ensuring the cultural validity and integrity of the study outcomes [13]. Employing a qualitative approach provided the space for us to explore and understand how Tongan males make sense of their experiences of gambling in New Zealand and the homelands. There is a significant body of quantitative and anecdotal reports about Tongan gambling behaviours [2]; generally, however, there has been little in-depth qualitative research on this topic [6]. While quantitative methods can enable the research to get a broad understanding of a phenomenon, qualitative approaches are able to delve into complex processes and illustrate the multifaceted nature of human phenomena [14].

The phenomenological approach was viewed as the appropriate paradigm for this study. Patton [15] writes that phenomenology is a way of exploring “how they [participants] perceive the phenomenon, describe it, feel about it, judge it, remember it, make sense of it, and talk about it with others” (p. 104). We employed a cultural research tool, talanoa, to collect and share perceptions and experiences. The purpose of the talanoa research method was to bring stories together in the Tongan norm of fono (meeting) and in a process of the co-construction of knowledge, which is the Pacific way [16]. Talanoa should never be mistaken for merely talking or having a discussion [17]. It can be complex and multi-layered and can range from free to critical discussion [18]. The discussion is not bound by having to remain within the two-way process of question and answer. Talanoa is best conducted in the language of the people being interviewed as this facilitates real understanding of both the content and the context of the dialogue and the research [17, 19].

### Participant recruitment

In order to get a spread by age, location, and experience, two key hubs, which are both spaces commonly used by Tongan males, were identified and used to recruit participants for this study—the church and the faikava.

Christianity and the church play a significant role in shaping Tongans’ cultural, social, spiritual, and economic life [20, 21]. Central to the spiritual life, the church has been described as a space not only for adults to hold on to and practice the Tongan culture but also where youth learn the characteristics and behaviour intrinsic to Tongan values and beliefs [22]. While gambling is often perceived as a negative practice by the church, churchgoers saw its value in protecting the financial and holistic well-being of their families. In fact, as the topic was being introduced, churchgoers acknowledged the importance of this sensitive issue being explored. The recruitment of potential participants from the church included several visits (by EF) to youth groups and services, and attendance at a church camp that addressed issues such as the prevention and reduction of social and health issues including alcohol and drug use, suicide, and gambling. Three Tongan Methodist and two Catholic churches gave EF an opportunity to speak about the study and invite potential participants to the talanoa. Participant information sheets were left with church leaders, along with contact details. As a result, two elders and two youth were recruited from church groups mentioned above.

The other recruitment site for this study was the kava space. Faikava is a well-known Tongan ceremonial, cultural practice that in recent times has been adopted as an informal and recreational activity embedded in the activities of some churches and other agencies in Tonga, and in Tongan migrant communities in New Zealand, Australia, and the USA [23]. As part of the recruitment process, EF approached and engaged in five faikava sessions around the Auckland region. Three faikava sessions were affiliated to churches, namely the Tongan Methodist Church of New Zealand, The Church of Latter-Day Saints, and the Catholic Church; the two remaining faikava sessions were with a community-based group and an old-boys’ association. These visits to the faikava sessions required the building of a rapport and having the ability to relate to others in a way that creates a level of trust. Participant information sheets were also provided to the kava clubs. Through the faikava sessions, the elders recommended other potential participants from their respective churches to participate in this study. From that, six elders were recruited from other kava clubs across Auckland. Two elders were recommended by the elders from the Tongan Methodist Church to contact EF. Both elders were sent participant information sheets and agreed to participate in an FGT.

### Data collection

Both English and Tongan languages were used in the FGT and the individual talanoa as preferred by the Mātu’a and To’utupu to express their responses. Several Mātu’a in the individual talanoa used Tongan only. The Mātu’a FGT lasted from over three hours to over four-and-half hours. The To’utupu FGT lasted between two to over three hours. The Mātu’a and To’utupu individual talanoa varied between 45 min and two-and-half hours. The Mātu’a FGT took place in a local hall and a community centre. The To’utupu FGT took place at a university classroom or a local church hall. The Mātu’a individual talanoa took place in their homes, at a café or at a workplace. The To’utupu individual talanoa took place at a university office, workplace or café.

All FGT and individual talanoa started and closed with a lotu (prayer), which was led by individuals in the group. The application and influence of prayer, which provided the opportunity for participants to share their perceptions and experiences in a secure and safe space, was apparent for all FGT and individual talanoa.

Food is critical as a means of reciprocity and acknowledging Tongan people for the time and space given to this study. For Mātu’a and To’utupu, refreshments were provided and were often consumed prior and during the focus group and individual talanoa. This was vital in building and maintaining relationships as the sharing of food plays a significant role in tauhi vā (nurturing relationships) with Tongans (Fehoko, 2014).

Reciprocal respect was expressed in the form of gift vouchers. The majority of the Mātu’a and To’utupu shared their gratitude for the me’a’ofa (gift) and the refreshments. No Mātu’a and To’utupu were aware of the gift vouchers beforehand, to ensure that this provision would not influence their decision to be involved in this study. Because of this, the Tongan value of fetokoni’aki (reciprocal respect) was acknowledged in the sharing of in-depth knowledge, perceptions, experiences, and time that each Mātu’a and To’utupu added to this study.

The data collection for this study took almost eight months. The constant visits to the churches and faikava were a lengthy but critical process to ensure the trust of the participants.

### Transcriptions

Transcriptions of the FGT and individual talanoa sessions were written in the languages that were used during the talanoa (Tongan and English) and transcribed within 24–48 h. This was to ensure that the ideas, knowledge, and understandings obtained from the sessions were maintained. Draft copies of the transcriptions were sent to each participant to confirm that the content was accurate and complete, to understand a few proverbs or phrases that were expressed in the heliaki (metaphorical) language, and to further add any other comments or ideas. All Mātu’a and To’utupu participants confirmed that the transcripts were accurate and complete.

### Data interpretation

A thematic analysis approach was employed to analyse the data. Thematic analysis provided a highly flexible approach to gain a rich, detailed, and complex understanding of the talanoa [24]. Thematic analysis reports the experiences, the meanings, and the reality of the participants. Rubin and Rubin [25] proposed that analysis is an exciting process because “you discover themes and concepts embedded throughout your interviews” (p. 35). We found this to be true. Thematic analysis was also useful for summarising key features of the views shared in FGT and individual talanoa, and it provided us with a well-structured approach to considering and handling those views [26].

The first step in the interpretation of data required familiarity with the data, generating initial codes, and searching for, reviewing and naming themes. After transcribing the talanoa, this first step involved reading and re-reading the transcripts. During these readings, notes were made on the margins using a colour coding system to identify potential themes. The themes were then organised to make consistent and meaningful reports which added up to a valuable account of the fundamental nature of the Tongan male perceptions and experiences of gambling and gambling harm grounded in their own words [24]. Several themes were coherent, consistent, and distinctive amongst all FGT and individual talanoa. After re-reading all transcripts, the audio recordings of the talanoa were replayed to identify any further themes, ideas, experiences, and perceptions of the participants.

## Ethical approval

This study obtained ethical approval from the Auckland University Technology Ethics Committee (AUTEC) on 2 February 2017.

## Results

The key themes arising from the Tongan perceptions of gambling harm prevention and reduction can be grouped into four main areas: 1. Awareness of gambling harm providers; 2. Church-based influences; 3. Community-based strategies; and 4. Cultural-based approaches. These were the main themes raised through the FGT and individual talanoa when addressing the research question, focussing on key gambling harm reduction and prevention strategies from a Tongan male perspective.

### Sample size

The characteristics of the sample are shown in Table 1. In total, 46 males participated in this study, consisting of 28 Tongan male elders (Mātu’a) and 18 Tongan male youth (To’utupu). All elders were born in Tonga before migrating to New Zealand, and all youth were born and raised in New Zealand. A total of 22 elders participated in the focus group talanoa (FGT), with 10 elders in the first FGT and 12 in the second FGT. Six elders participated in the individual talanoa. In relation to age, of the six elders, two were in their 40s, one in their 50s, and three in the 60s. In terms of the preferred gambling activities of the 28 elders, five indicated that they had never engaged in gambling activities, 11 with betting (horses), four with lotto, two with casino games, and six with the EGMs (see Tables 1 and 2).

**Table 1:** The Mātu’a preferred gambling activity—focus group talanoa

Participants	Preferred gambling activity	Ages	Participants	Preferred gambling activity	Ages
Mātu’a 1.1	Betting (Horses)	60s	Mātu’a 2.1	EGMs	40s
Mātu’a 1.2	–	50s	Mātu’a 2.2	Betting (Horses)	60s
Mātu’a 1.3	Lotto	50s	Mātu’a 2.3	Betting (Horses)	40s
Mātu’a 1.4	–	60s	Mātu’a 2.4	Betting (Horses)	40s
Mātu’a 1.5	Betting (Horses)	50s	Mātu’a 2.5	Betting (Horses)	50s
Mātu’a 1.6	Casino	40s	Mātu’a 2.6	EGMs	50s
Mātu’a 1.7	EGMs	40s	Mātu’a 2.7	-	70s
Mātu’a 1.8	–	70s	Mātu’a 2.8	Lotto	60s
Mātu’a 1.9	Betting (Horses)	50s	Mātu’a 2.9	Lotto	40s
Mātu’a 1.10	EGMs	40s	Mātu’a 2.10	Betting (Horses)	40s
			Mātu’a 2.11	–	70s
			Mātu’a 2.12	Betting (Horses)	50s

**Table 2:** The Mātu’a preferred gambling activity—individual talanoa

Participants	Preferred gambling activity	Ages
Ma’ake	EGMs	40s
Maika	Lotto	40s
Mote	Betting (Horses)	50s
Misi	Betting (Horses)	60s
Mone	Casino	60s
Miu	EGMs	60s

Mātu’a’ 1.1 refers to the first group in the first participant. Mātu’a’ 2.1 refers to the second group and first participant. Similar process is evident with the To’utupu table below.

Pseudonyms were used to protect the identity of the participants involved in the individual talanoa for both the Mātu’a’ and To’utupu. Twelve youth participated in the FGT with four in the first FGT and eight in the second FGT, while six youth participated in the individual talanoa. In relation to age, two youth participants were in their late teenage years, eight in their 20s, and two in their 30s. Two youth shared how they had never engaged in any gambling activity, seven youth liked EGMs, eight engaged with betting (sports), and one with casino games (see Tables 3 and 4).

**Table 3:** The To’utupu preferred gambling activity—focus group talanoa

Participants	Preferred gambling activity	Ages	Participants	Preferred gambling activity	Ages
To’utupu 1.1	EGMs	30s	To’utupu 2.1	Betting (Sports)	20s
To’utupu 1.2	Betting (Sports)	20s	To’utupu 2.2	EGMs	20s
To’utupu 1.3	Betting (Sports)	20s	To’utupu 2.3	–	18–20
To’utupu 1.4	EGMs	20s	To’utupu 2.4	Betting (Sports)	30s
			To’utupu 2.5	EGMs	20s
			To’utupu 2.6	Casino	20s
			To’utupu 2.7	–	18–20
			To’utupu 2.8	Betting (Sports)	20s

**Table 4:** The To’utupu preferred gambling activity—individual talanoa

Participants	Preferred gambling activity	Ages
Tevita	Betting (Sports)	20s
Toni	EGMs	30s
Taani	EGMs	20s
Taniela	EGMs	20s
Tui	Betting (Sports)	30s
Tika	Betting (Sports)	20s

### Theme 1: “awareness of gambling harm providers”

The majority of the participants were not aware of gambling treatment providers. Furthermore, almost all said they had no knowledge about what Pacific gambling treatment providers actually offered to Tongan families and communities affected by problem gambling. Some said they would be appreciative if translated versions of information regarding problem gambling were in Tongan.

*“I am not aware of any gambling treatment providers in our [Tongan] community or know of anyone who would seek help. I think more people get their support and resources from the church.” [Misi]*

*The one Tongan elder who self-identified as a problem gambler did not seek any support from a gambling treatment provider regarding his behaviour. Similar experiences were also shared by Tongan youth, with none seeking support from a gambling treatment provider. This may also be due to no Tongan youth experiencing problem-gambling behaviours or feeling comfortable with seeking support because of being unfamiliar with what gambling treatment providers offer young people.*

### “Bro, I don’t know of any groups that help our gamblers, particularly for our Pacific community.” [To’utupu 1.4]

It is important to acknowledge that the development of “Pacific for Pacific” services is not the only answer to improving care for Pacific people. One Tongan participant suggested, gambling treatment service providers, whether Pacific or non-Pacific, need to offer a holistic approach to supporting families as well:

*“I haven’t heard of a specific service that could help our Tongan gamblers. Like I have heard of the alcohol and drugs services but not so much gambling ones … we need a holistic approach.” [Mone]*

### Theme 2: “the influence of church on gambling harm prevention and reduction”

Some Tongan elders argued that the church is an undervalued but critical space to speak about the need to prevent and minimise gambling harm. Tongan church leaders need to identify and address the health and social issue of gambling harm. Further, Tongan churches need to be equipped with current research on these issues:

*“We need a framework that can address these issues where spirituality is the core. If the messages are not coming from the pulpit, then how can we address the issue for that is the most powerful and influential place.” [Mātu’a 1.8, Non-Gambler]*

As indicated by several participants, churches during Easter weekends would carry out Easter camps, dedicated to the whole congregation addressing social issues such as alcohol consumption, drug use and abuse, and suicide. Most recently, gambling has been introduced as a health and social issue.

Because of this, the majority of the Tongan youth who participated in the study are encouraging churches to continue to promote messages associated with gambling harm prevention and reduction:

*“I think the only programmes that I have heard of, and I know that is not working properly, is the church camp that it is all about prevention of alcohol consumption and drug use.” [To’utupu 2.6]*

Some Tongan elders shared that some church leaders have limited knowledge around gambling harm. Further, several participants indicated that there are educated people, including health professionals, in congregations who have directly experienced gambling harms or who are trained to help others, and these people could be encouraged to connect with other members of the congregation:

*“I think it is important that our church leaders acknowledge that members of the church are also health professionals who work in the health field and sector. Our people in the field of health and church is a win for all.” [Maika]*

However, whilst the church continues to address issues around gambling, the majority of Tongan elders suggested that gambling treatment providers need to collaborate with churches to identify cultural strategies and spiritual interventions for Tongans by Tongans.

### Theme 3: “community-based strategies in addressing gambling harm behaviours”

Almost all the participants noted that Electronic Gaming Machines (EGMs) are widely accessible and available in sports bars and clubs around New Zealand, and in South Auckland. Whilst there was no mention of reducing the access to and availability of horse betting venues and EGMs in the casino and sports bars, all Tongan elders and youth in FGT and individual talanoa were in agreement about reducing the number of EGMs in prominent gambling venues:

*“Fastest way [to reduce EGMs] is to get rid of the machines especially in my area [South Auckland] because that’s what create the problems for our people.” [Mātu’a 2.2]*

*“Because I know they won’t take it away but it would be good to put some measures in place around cutting down people’s access to it so if I was to start like in South Auckland I’d take some out; don’t renew it.” [Mātu’a’ 2.9]*

There was a lack of knowledge from both Tongan elders and youth perspectives in regard to what gambling policies and regulations were. However, they proposed health professionals and government officials should introduce and embed awareness around gambling and gambling harm in the education curriculum. Engaging and participating in social games at school that consist of gambling elements and behaviours led to the transition to gambling activities such as EGMs in the casino and sports betting. Several Tongan youth commented on the need for parents and schools to have a critical talanoa on the transition from social games in schools to gambling activities such as sports betting and EGMs in the casino:

*“I think there is a desire of young people to learn about the consequences around gambling and understanding the deeper issue around gambling in schools which I think is more important than some of the existing subjects today [laugh] … especially if we are in a generation where social games can lead to other mental health and addiction problems that we see on the media.”[To’utupu 1.4]*

All Tongan youth in the second FGT were recruited from outside the South Auckland region. Not surprisingly, several To’utupu indicated that there is a lack of presence of Pacific gambling harm treatment providers in other areas in Auckland. Consequently, some To’utupu commented that whilst most Tongans reside in South Auckland, Pacific gambling harm treatment providers need to take into consideration other Tongans across Auckland:

*“We as Tongans need more than one session where they come and run a gambling workshop. Pacific gambling services need to be more active not only in South Auckland but also around the Auckland region; just because many Tongans are out South does not mean they ignore us.” [To’utupu 2.3]*

Some To’utupu indicated the significant increase in advertisements for gambling opportunities across social media platforms, sporting events, TV programmes, and billboards compared to the lack of advertisements of gambling harm support and treatment services. In contrast, several To’utupu raised the need to increase the awareness of gambling treatment providers across all media and social media platforms and sporting events:

*“To be honest and thinking about it now, I have not heard of any preventative programmes on gambling. I have seen some of the ads you know like if you are friend that shows these signs call this number, gambling helpline number, and some other stuff but other than that, I think there are more promotion of gambling campaigns than preventative of gambling campaigns, which is quite sad.” [To’utupu 1.3]*

### Theme 4: “cultural strategies addressing gambling harm behaviours”

The space of the faikava for Tongan males was said to be valuable and culturally safe and secured space for ideas, perceptions, and lived experiences to be shared in a democratic way [20, 28]. For example, one To’utupu indicated he had support and words of advice from a counsellor who had worked at a gambling treatment provider, and who had engaged with him at a faikava:

*“I believe the faikava will be good spot for people working in gambling services to come and address the Tongan male because it is the space where you can find a lot of them you know.” [To’utupu 2.6]*

More important to this study is the need for gambling treatment providers to explore cultural and traditional practices, like the faikava, as a medium which addresses this social and health issue. In fact, the significance of the faikava is underpinned by the importance of socialising, sharing, and communicating in a harmonious way without the fear of being judged or misinterpreted. Another key finding was identifying cultural practices like the faikava as a social and cultural space to disseminate problem-gambling prevention and reduction resources and information. The majority of the Tongan elders and youth suggested that the faikava is an appropriate practice for Tongan males to discuss Tongan male and community issues, one being gambling and problem-gambling behaviours.

*“There should be a space where the two generations [elder and young] can talk. That way, there is a connection and relationship happening you know.” [To’utupu 2.1]*

Interestingly, several participants put forward the need to reassess and re-align church policies and constitutions to fit the New Zealand lifestyle and to address contemporary issues that may have been absent in the homelands, one being gambling. For example, as a few of the Tongan elders noted, the rolling of dice and playing cards are defined as gambling under the constitution of the Wesleyan Church, which has the largest number of churchgoers in Tonga and a significant number across New Zealand, Australia, and the USA. Similar ideas were also proposed by some of the Tongan youth arguing that churches need to be accountable for subconsciously promoting and encouraging people to engage in gambling through church services. It is important that churches critically re-evaluate their policies to address ways of preventing and minimising problem-gambling behaviours by adding gambling activities such as casino, EGMs, horse-race betting, Lotto, and bingo to the list of forbidden gambling practices.

*“You have to address the belief systems of the mind, body and soul of our people. I think that is where services need to work together with the church, youth groups and church leaders. That could be the spiritual intervention can work and expose the evil in gambling. … I think regarding the services, they are just applying a Band-Aid instead of going deep to address the issue.” [Miu]*

## Discussion

This paper explored the views of Tongan males of how to prevent and reduce gambling harm amongst the Tongan community in New Zealand. Three key themes were identified which include increasing the awareness of gambling harm treatment providers, influencing religious leaders to address gambling harm in churches, exploring community-based strategies and utilise cultural practices as a medium to disseminate gambling harm information and resources.

Previous research found that males would often share their perceptions and experiences in an environment that is usually without their close relatives [20]. This is due to the sensitivity of the talanoa that may be shared, which goes against cultural values and protocols, particularly the relationship between brothers and sisters and father and children.

The interface of culture and religion plays an integral role in and across many Tongan societies. Tongan religious leaders are well-respected individuals in the Tongan social structure and play a vital role in sharing information with Tongan people.

Previous research has illustrated that some Pacific people have concerns regarding accessing care or support from a Pacific worker, due to the small size of the Pacific community and the risk of confidentiality being jeopardised [12]. The majority of the Tongan elders in the present study also commented on how there is “information overload” on alcohol consumption and drug abuse, but a lack of resources regarding gambling and gambling harm. Furthermore, the majority of the Tongan elders proposed that church leaders and Pacific gambling treatment service providers should work in partnership in an attempt to inform churchgoers about the issue around gambling and gambling harm.

As a result, Tongan males may not seek support from a gambling treatment provider because they view their gambling as “fun” and “social”. A recent study on older Chinese migrants in New York shared similar views, with participants indicating that support systems are critical for fun and social gamblers, which would prevent an increase in the number of problem gamblers in the future [29]. Moreover, the majority of the participants in this study suggested that gambling treatment providers need to collaborate more effectively with traditional Tongan churches, who may resist discussing gambling and problem-gambling issues. To that point, several participants acknowledged the role of church camps in addressing the social and health issue of gambling and problem-gambling behaviours. Other issues addressed in that context include suicide prevention, and alcohol and drug use and abuse.

Tonga is a hierarchical society with the father as the head of the family. As a result, this structure creates communication barriers, in particular between children and parents [30, 31], creating intergenerational conflict [20, 22]. Consequently, to address these communication barriers, there is a need for gambling treatment providers to explore cultural and traditional practices, like the faikava, as a medium to address this social and health issue. In fact, the significance of the faikava is underpinned by the importance of socialising, sharing, and communicating in a harmonious way without the fear of being judged or misinterpreted. Another key finding was identifying cultural practices like the faikava as a social and cultural space to disseminate gambling harm prevention and reduction resources and information. The majority of the participants suggested that the faikava is an appropriate setting for Tongan males to discuss Tongan male and community issues, one being gambling and problem-gambling behaviours.

More important to this study is the need for gambling treatment providers to explore cultural and traditional practices, like the faikava, as a medium in which to address this social and health issue. In fact, the significance of the faikava is underpinned by the importance of socialising, sharing, and communicating in a harmonious way without the fear of being judged or misinterpreted. Another key finding was identifying cultural practices like the faikava as a social and cultural space to disseminate problem-gambling prevention and reduction resources and information. The majority of the Tongan elders and youth suggested that the faikava is an appropriate practice for Tongan males to discuss Tongan male and community issues, one being gambling and problem-gambling behaviours. Gambling treatment providers must also consider adding culturally safe initiatives and programmes in order to encourage Pacific people to seek their support services. Furthermore, the importance of establishing relationships “for Pacific-by-Pacific people” will support gamblers in seeking gambling treatment providers to address their problem-gambling behaviours.

### Limitations

This study is not without limitations. Firstly, the study was conducted with participants from a single urban centre, namely Auckland, New Zealand. This was believed to be appropriate because the majority of the New Zealand Tongan population live in the Auckland region [32]. Secondly, as this study focussed solely on the Tongan male experiences and perceptions of gambling and gambling harm in New Zealand, the Tongan female voice was missing. Thus, further studies are needed to explore the Tongan female elders and female youth meanings and understandings of gambling and gambling harm within the ambit of the family.

## Conclusions

This study’s findings highlight a number of factors which must be taken into account in the development of public health programmes for Pacific and Tongan people in New Zealand for preventing and reducing gambling harm. These include the need to increase the awareness of gambling harm providers in communities, a greater influence with the church and church leaders, exploring community-based strategies, and using cultural practices like the faikava to promote gambling harm prevention and reduction from a Tongan male perspective. More qualitative studies through Pacific worldviews, methodologies and methods would fully capture Pacific meanings in explaining why Pacific people engage in gambling to the extent of being at a greater risk of developing problem-gambling behaviours than the general population.

## Data Availability

The data analysed in this study are not publicly available.
